# Temporal Artery Flow Response during the Last Minute of a Head Up Tilt Test, in Relation with Orthostatic Intolerance after a 60 Day Head-Down Bedrest

**DOI:** 10.1371/journal.pone.0022963

**Published:** 2011-10-26

**Authors:** Philippe Arbeille, Ming Yuan, Yanqiang Bai, Shizhong Jiang, Gullemette Gauquelin, Patrick Aubry, Yuming Wan, Marc Antoine Custaud, Yinghui Li

**Affiliations:** 1 UMPS-CERCOM Médecine Physiologie Spatial, Université-Hôpital Trousseau, Tours, France; 2 The State Key Laboratory of Space Medicine Fundamentals and Application, China Astronaut Research and Training Center, Beijing, China; 3 Centre National ‘d’Etudes Spatiales, Paris, France; 4 UMR CNRS 6214 – INSERM 771, Faculté de Médecine d'Angers, Angers, France; Institut Pluridisciplinaire Hubert Curien, France

## Abstract

**Objective:**

Check if the Temporal flow response to Tilt could provide early hemodynamic pattern in the minutes preceding a syncope during the Tilt test performed after a 60-d head down bedrest (HDBR).

**Method:**

Twenty-one men divided into 3 groups [Control (Con), Resistive Vibration (RVE) and Chinese Herb (Herb)] underwent a 60 day HDBR. Pre and Post HDBR a 20 min Tilt identified Finishers (F) and Non Finishers (NF). Cerebral (MCA), Temporal (TEMP), Femoral (FEM) flow velocity, were measured by Doppler during the Tilt. Blood pressure (BP) was measured by arm cuff and cardiopress.

**Results and Discussion:**

Four of the 21 subjects were NF at the post HDBR Tilt test (Con gr:2, RVE gr: 1, Herb gr: 1). At 1 min and 10 s before end of Tilt in NF gr, FEM flow decreased less and MCA decreased more at post HDBR Tilt compared to pre (p<0.05), while in the F gr they changed similarly as pre. In NF gr: TEMP flow decreased more at post HDBR Tilt compared to pre, but only at 10 s before the end of Tilt (P<0.05). During the last 10 s a negative TEMP diastolic component appeared which induced a drop in mean velocity until Tilt arrest.

**Conclusion:**

The sudden drop in TEMP flow with onset of a negative diastolic flow preceding the decrease in MCA flow confirm that the TEMP vascular resistance respond more directly than the cerebral one to the cardiac output redistribution and that this response occur several seconds before syncope.

## Introduction

Many astronauts after exposure to real (spaceflight) or simulated (HDBR: Head down bedrest) microgravity, experience orthostatic intolerance, become hypotensive and presyncopal when they assume an upright position. Orthostatic intolerance may interfere with astronaut function during reentry and after spaceflight and may limit the ability of an astronaut to exit a landed spacecraft unaided during an emergency. Thus, it is very important to evaluate the orthostatic tolerance for each crewmembers in order to optimize the counter measure designed for improving their tolerance. The methods for evaluating orthostatic tolerance include stand and tilt test, and lower body negative pressure (LBNP). Presently the decision to interrupt an orthostatic test is made on the basis of the clinical signs of intolerance, the heart rate, and the blood pressure (BP, arm cuff and Cardiopress). Previous HDBR showed that the most frequent and reliable sign preceding the onset of syncope is the drop in blood pressure but in some cases the cerebral flow velocity dropped some cycles earlier or in parallel with the BP drop, or a bradycardia preceded the BP drop by some seconds [1]–[3].

Nevertheless even in case of non efficient cardiac output redistribution among the major vascular territories (Splanchnic and lower limb) due to a insufficient reduction of flow volume in both area [4] the cerebral flow velocity does not drop immediately due to the autoregulation [1], [2], [5] which could explain why cerebral flow drops lately when all compensatory process have been triggered.

Our hypothesis was that the Temporal flow measured at the same level of altitude (from the heart) as the cerebral one during a stand or tilt test should be affected by the abnormal cardiac output redistribution earlier than the cerebral flow because it is not as acutely protected as the cerebral one by the autoregulation process. The objective of the present study was to monitor simultaneously and continuously the cerebral and temporal flows by Doppler during the last minute of a Tilt test before and after a 2 months bedrest.

## Methods

Twenty one healthy male subjects participated in the 60-day -6 degree HDBR at China Astronaut Research and Training Center (ACC, Beijing, China). Before HDBR, the subjects were aged 30.4±0.9 years, with average weights and heights of 59.9±0.9 kg and 168.5±0.7 cm, respectively. None had any history of cardiovascular or other major disease and all underwent an extensive medical examination before being included in this study. All the subjects received a complete description of the experimental procedure before giving their written informed consent to the protocols conformed to Helsinki declaration and approved by the ethical committee of China Astronaut Research and Training Center. The subjects were randomly assigned into control group “Con gr” (HDBR without counter measure, n = 7), HDBR with daily Chinese herbs consumption (Herb gr, n = 7) and HDBR with daily resistive vibration exercise (RVE gr, n = 7).

### HDBR program

The study consisted of a 15 day ambulatory control period, 60 d HDBR and 24 d recovery period. During the bed rest, the subjects remained **−**6 degree head-down tilt position continuously except a daily 10 minutes stand for hygienic procedure and weighting. Coffee, tea and smoking were prohibited all along the experiment. The subjects were supervised and monitored 24 h per day. Room lighting was on between 6:30 AM and 22:30 PM. The subjects in Herb group were given orally 6 g pills, 3 times daily. The placebo was given orally to control subjects based on double-blind principle. The subjects in RVE group were exposed to one daily session of resistive vibration exercise for 24 minutes according to the training protocol, i.e, each session consisted of five stretches, with 4 minutes RVE each and with 1 minute rest. The characteristics of the RVE platform scenario were the following: 30 Hz vibration, resistive loads of 1.5 times of the body weight, magnitude of 0.3 g peak-to-peak, and amplitude <0.1 mm.

### Tilt test scenario

Post HDBR 20 min tilt test identified subjects as Finishers (F) and Non Finishers (NF). The tilt test consisted of 10 min resting supine for instrumentation followed by 10 min of resting measurements, then a 75 degree head up tilt for a maximum of 20 min. During these tests, HR was obtained by 3 lead electrocardiography (Milwaukee, Wisconsin, USA) and SBP and DBP were measured continuously with Cardiopress, a non-invasive finger cuff method (Biomedical Instrumentation TPD-TNO, Amsterdam, Netherlands). A height corrector placed at the level of the heart provided a stable measurement independent of hand position supine or tilting. The test was ended if one of the following signs occurred: pre-syncope symptoms (nausea, clammy skin, excessive sweating, pallor, vertigo), SBP fall >25 mmHg/min or DBP fall>15 mmHg/min, a SBP<70 mmHg, HR fall>15 beats/min, cardiac dysrhythmias).

### Echographic and Doppler Measurements

During the TILT test, the Cerebral flow velocity (MCA Flow) was recorded using a 2 MHz transcranial Doppler probe fixed over the temporal window to insonate the right middle cerebral artery (MCA). The angle of insonation of the MCA was considered to be 0 degrees. The Temporal flow velocity (TEMP) was recorded using a Doppler probe (4 MHz) placed in from of the Temporal artery at the level of the upper and anterior part of the left ear .

The superficial femoral artery flow velocity (FEM) was investigated using a flat Doppler probe of 4 MHz fixed by 2 straps passing around the upper part of the thigh and around the abdomen. The Doppler beam was steered at 45 degree from the front face of the probe and the angle between the Doppler beam and the vessel axis, remained unchanged during the session. The Doppler spectrum was recorded and analysed by the Cardiolab ground module (CNES device France). Based on an earlier report [6] it was assumed that the diameter of these vessels remained constant during the Tilt test and that mean velocity changed in proportion with flow volume (ml/min) as calculated from velocity and vessel cross section. The Cerebral, Temporal, Femoral flow change were evaluated through the corresponding mean velocities. The Femoral systolic velocity (FEM S) was measured as the peak velocity on the Femoral Doppler spectrum. From these Cerebral, Temporal, Femoral mean velocities we calculated the Cerebral to Femoral flow ratio [CFR =  (MCA flow/FEM flow)], and the Temporal to femoral flow ratio (TEMP flow/FEM flow) which measure the proportion of flow supplying the leg and the brain or temporal areas.

### Parameters display

Cerebral (MCA), Temporal (TEMP), Femoral (FEM) mean flow velocity, and Femoral systolic velocity (FEM S) were measured continuously by Doppler during the Tilt. Blood pressure (BP) was also measured by arm cuff and Cardiopres. All these parameters were measured at 3 min pre Tilt in supine position, at **−**1 min, and **−**10 s before the end of the test. The percent changes were displayed as mean+/**−**SD.

### Statistical Analyses

Tilt data analysis: The percent change from supine to 1 minutes before the end of the Tilt and the last 10 s of the Tilt were analyzed with the data grouped according to whether the subjects finished (F) the post-HDT 20-min Tilt test or not (NF). Data were expressed as mean ± SD. Statistical comparison were performed using a one way repeated measures analysis of variance SAS 9.1.3 analysis software (Cary, NC, U.S.A.) with main effect of group (F or NF) at post-HDT 20-min Tilt. Differences were considered as significant for P<0.05.

## Results

**Figure 1:**
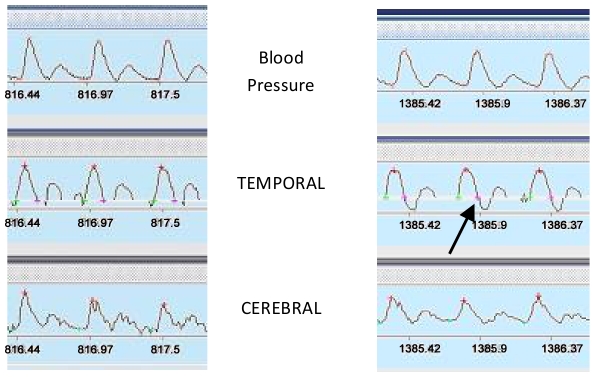
BP trace and TEMP and MCA normal flow velocity (at −1 min) on left panel. BP, MCA normal flow velocity, TEMP with negative diastole at **−**10 s before tilt arrest (arrow) on right panel.

**Figure 2:**
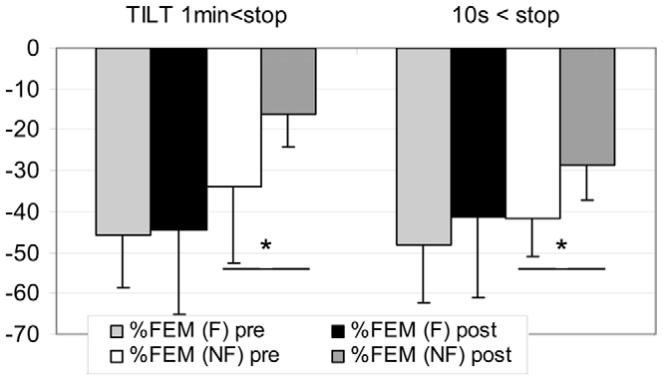
Percent decrease in FEM (femoral) flow at 1 min and 10 s before end of Tilt (from supine) in F (finisher n = 17) and NF (non finisher n = 4). Similar change pre/post HDBR at 1 min and 10 s. (mean+/**−**SD, * P<0.05)

**Figure 3:**
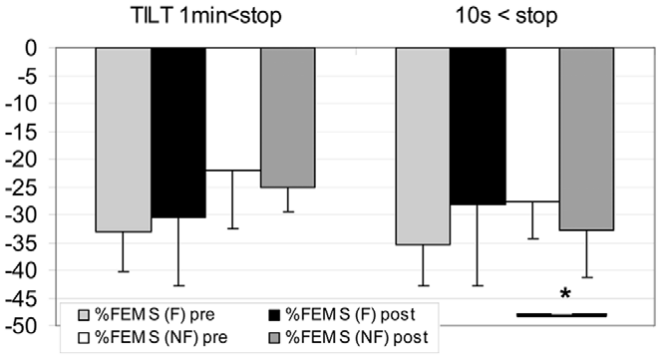
Percent decrease in FEM S (Femoral Systolic flow velocity) at 1 min and 10 s before end of Tilt (from supine) in F (finisher n = 17) and NF (non finisher n = 4). FEM S change proportionately with Aortic flow ie stroke volume. In F gr similar change pre/post HDBR at 1 min and 10 s. In NF gr Post HDBR FEM S flow velocity at 10 s before Tilt arrest drops more than pre HDBR (mean+/**−**SD, * P<0.05)

At post HDBR Tilt test, 4 of the 21 subjects were NF (Con gr: 2, RVE gr: 1, Herb gr: 1) – During the last 10 s of the Tilt the TEMP flow velocity pattern in the 4 NF changed from forward flow in systole and diastole to forward flow in systole but negative flow in diastole while the MCA and FEM flow pattern did not (Figure 1). In the NF gr: FEM flow decreased less at post HDBR Tilt compared to pre, both at 1 min (pre: **−**34±19% vs post: −16±8%) and 10 s (pre: −42±9% vs post: −29±9%) before the end of the Tilt (P<0.05). In the F gr, FEM flow decreased both at 1 min and at 10 s, but similarly at pre and post HDBR Tilt (Figure 2). In the NF gr: FEM S decreased similarly at pre and post HDBR at 1 min, but decreased significantly more at post HDBR in the last10 s (P<0.05) compare to pre (Figure 3). In the F gr FEM S decreased similarly both at 1 min and 10 s, at pre and post HDBR Tilt.

**Figure 4:**
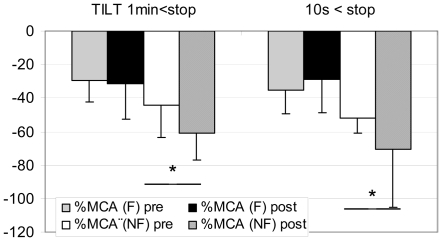
Percent decrease in MCA (Cerebral) flow at 1 min and 10 s before end of Tilt in F (finisher n = 17) and NF (non finishers n = 4). Similar change pre/post HDBR at 1 min and 10 s. (mean+/**−**SD, * P<0.05)

**Figure 5:**
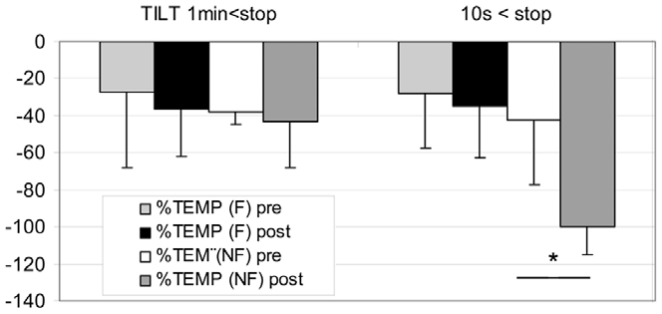
Percent decrease in TEMP (Temporal) flow at 1 min and 10 s before end of Tilt in F (finisher n = 17) and NF (non finisher n = 4). Post HDBR TEMP flow drops 10 s before Tilt arrest. (mean+/**−**SD, * P<0.05)

**Figure 6:**
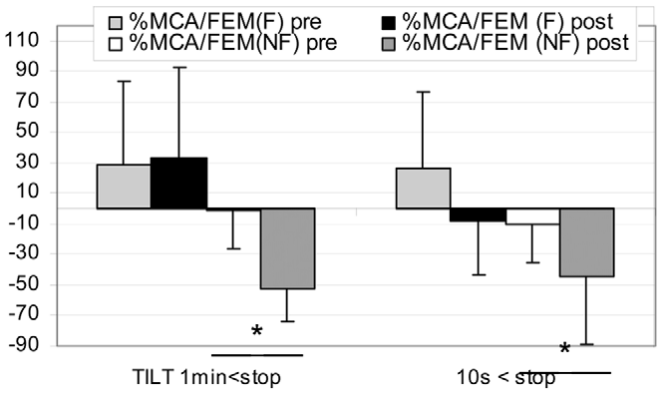
Percent change in Cerebral/Femoral flow ratio at 1 min and 10 s before end of Tilt in F (finisher n = 17) and NF (non finisher n = 4). Similar change pre and post HDBR at 1 min and 10 s. (mean+/**−**SD, * P<0.05)

**Figure 7:**
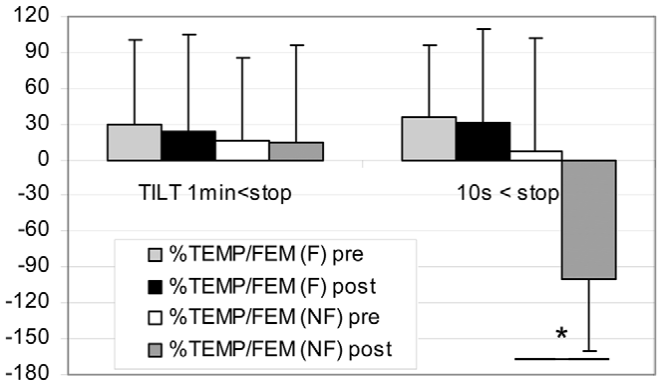
Percent change in Temporal/Femoral fow ratio at 1 min and 10 s before end of Tilt in F (finisher n = 17) and NF (non finisher n = 4). The ratio drops only 10 s before Tilt arrest (post HDBR), while on Fig. 2, FEM is similarly decreased at 1 min and 10 s. (mean+/**−**SD, * P<0.05)

In NF gr MCA flow decreased more at post HDBR Tilt compare to pre, both at 1 min (pre: **−**44±19% vs post: **−**61±16%) and 10 s (pre: **−**52±9% vs post: **−**70±35%) before the end of the Tilt (P<0.05). In F gr, MCA flow decreased similarly at pre and post HDBR Tilt both at 1 min and 10 s (Figure 4) - In NF gr: TEMP flow decreased more at post HDBR Tilt compared to pre, but only at 10 s (pre: **−**42±35% vs post: **−**100±15%) before the end of Tilt (P<0.05). During the last 10 s the diastolic component disappeared and a negative flow (reverse) appeared which induced the drop in mean flow velocity (Figure 1). Moreover, BP drop occurred after the onset of negative diastolic flow on the TEMP flow velocity pattern in the NF subject. In F gr, TEMP flow decreased similarly at pre and post HDBR Tilt (Figure 5) - In NF: MCA/FEM ratio decreased more post HDBR both at 1 min (pre: 1±26% vs post: –52±23%) and 10 s (pre: **−**10±25% vs post: **−**44±44%) before the end of Tilt, compare to pre HDBR Tilt (P<0.05). In F gr, MCA/FEM changed similarly at pre and post HDBR Tilt both at 1 min and 10 s before Tilt end (Figure 6) - In NF: TEMP/FEM ratio decreased more at post HDBR Tilt, only at 10 s (pre: 7±95% vs post: **−**100±60%) before the end of Tilt, compare to pre HDBR Tilt (P<0.05). In F gr TEMP/FEM changed similarly at pre and post HDBR Tilt both at 1 min and 10 s before Tilt end (Figure 7).

## Discussion

The counter measures had no significant effect on the orthostatic tolerance as the NF subjects were from the Con gr (n = 2) RVE gr (n = 1) and Herb gr (n = 1).

In the NF gr (a) FEM velocity reduced less than pre HDBR because of insufficient leg vaso-constriction after HDBR (b) MCA velocity reduced more because of less efficient cardiac output redistribution as confirmed by insufficient reduction in lower limb (femoral) and probably insufficient splanchnic flow reduction [4] (c) TEMP velocity decreased markedly post HDBR as a consequence of cardiac output drop and/or increased local vasoconstriction (d) around 10 s before the subject fainted, TEMP flow pattern changed (negative flow appeared) and flow velocity dropped compare to 1 min, while the FEM and MCA velocities remained at the same level as at 1 min.

Decrease in MCA/FEM means insufficient flow redistribution towards the brain, but does not signified that the subject is about to faint as there was no difference in MCA/FEM response from 1 min to 10 s before end of Tilt. Previous studies have shown that this ratio increased less during LBNP or Tilt tests at the end of bedrest or spaceflight in non finisher which means that the peripheral flow regulation was altered but no significant change was found in the last minute of these tests. [7], [8], [9].

Conversely TEMP/FEM ratio indicated several seconds before fainting an opposite change compare to pre HDBR, thus the TEMP flow velocity and TEMP/FEM ratio are earlier indicator of fainting than MCA and FEM velocities, and MCA/FEM ratio.

We suggest that the earlier drop in TEMP flow velocity is related to the fact that this area is not as accurately controlled as the MCA one (No autoregulation) and thus respond more directly to changes in cardiac output or other regional flows. Moreover the TEMP vascular resistance should increase in order to favour flow towards the brain which should contribute to reduce TEMP flow. Additionally the change in TEMP flow Doppler trace (negative flow) and the drop in TEMP mean flow are new and valuable parameters for anticipating the syncope several second prior to BP or MCA velocity drop. In fact the change in MCA and Temporal flow belong to the same mechanism: increase in vascular resistance in the territory they supply. The main advantage of the Temporal hemodynamic is that it makes the increase in vascular resistance easy to detect (reverse flow onset) and confirm a sudden and higher change in vascular resistance compare to the cerebral one. Moreover the increase in TEMP vascular resistance was more visible as there was no opposite process like autoregulation that could compensate partially for such increase. In the present study only 4 subjects did not finish their Tilt test, and showed reverse TEMP flow, but none of the 17 remaining Finishers showed a TEMP negative flow. The kinetic of Cerebral and Temporal flow change has to be evaluated on a larger number of subjects but the assessment of the TEMP hemodynamics provides additional information on the cardiac output redistribution during orthostatic test.

A previous Bedrest study with TILT+LBNP orthostatic test reported that the Systolic Femoral flow velocity changed in proportion with the Aortic flow volume in one cycle (ie SV) [10]. Thus the Femoral systolic velocity (FEM S) percent change represent in fact the change in SV in percent from supine. In the present study SV decreases similarly pre and post HDBR at 1 min in the F and NF gr. Conversely at 10 s prior to syncope FEM S (SV) dropped more compare to 1 min in the NF gr at post HDBR and significantly more compare to pre. Thus %SV decrease at 10 s prior to syncope at post HDBR should contribute to %MCA decrease but the additional drop in %SV (approx 8% more than pre or than 1 min post) is not sufficient at all to explain the marked additional drop of the %TEMP flow (approx 60% more than pre and than 1 min post). Moreover we can reasonably consider that the lesser decrease in FEM flow at 10 s contribute to reduce SV. At last the drop in TEMP flow and increase in TEMP vascular resistance may be related both to the additional SV drop and additional vasoconstriction in the TEMP area.

In summary and as already observed in previous studies [9], [10], the disadapted vascular response during orthostatic test is related to a multifactorial process which include drop in cardiac output, lack of leg and splanchnic vasoconstriction, reduced venous return from leg vein… It also confirmed that none of these processes plays the major role in the development of orthostatic hypotension. On the other hand these observation emphasizes the interest of the Temporal vascular response for evaluating the level of vasoconstriction which seems to be mostly dependent on the peripheral vasoconstriction process and for predicting the onset of syncope.
